# High-temporal velocity-encoded MRI for the assessment of left ventricular inflow propagation velocity: head-to-head comparison with Color M-mode echocardiography

**DOI:** 10.1186/1532-429X-17-S1-Q38

**Published:** 2015-02-03

**Authors:** Emmeline Calkoen, Nina Ajmone Marsan, Jeroen J Bax, Pieter J van den Boogaard, Arno Roest, Albert de Roos, Jos J Westenberg

**Affiliations:** 1 Pediatric cardiology, Leiden University Medical Center, Leiden, Netherlands; 2 Cardiology, Leiden University Medical Center, Leiden, Netherlands; 3 Radiology, Leiden University Medical Center, Leiden, Netherlands

## Background

Left ventricular (LV) inflow propagation velocity (Vp) is a useful parameter used in the complex assessment of LV diastolic function and is measured by Color M-mode echocardiography. The aim of current study was to develop an alternative method for Vp-assessment using high-temporal single-directional velocity-encoded Magnetic Resonance Imaging (VE-MRI).

## Methods

**Figure 1:**
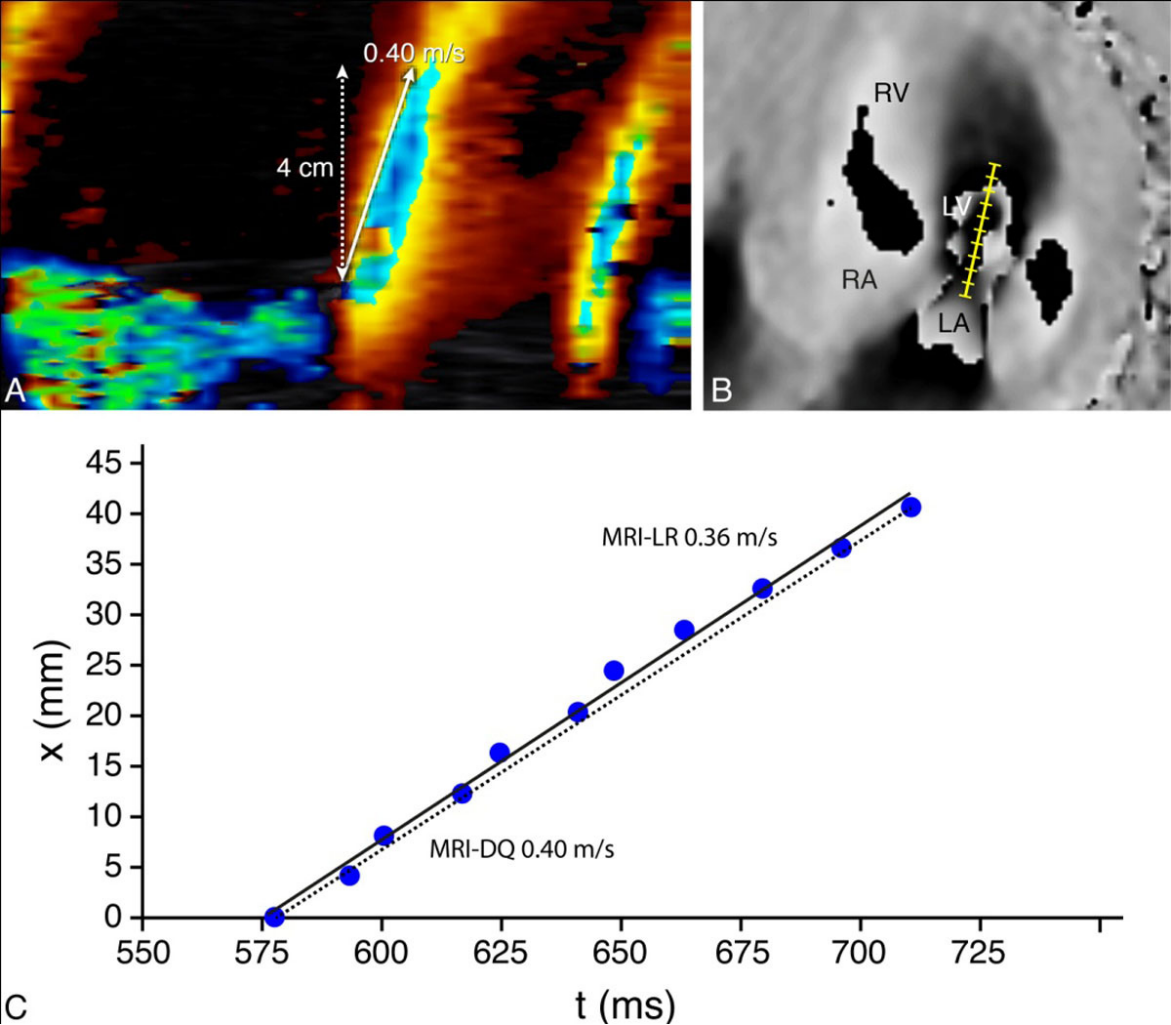
Vp assessment with Color M-mode echocardiography (panel A) and velocity-encoded MRI (panel B). The position-time graph (panel C) with Vp-MRI-Difference-Quotient (dotted line) and Vp-MRI-Lineair-Regression (solid line).

43 patients with ischemic heart failure (61±11years) and 22 healthy volunteers (29±13years) were included. Vp by Color M-mode echocardiography (Vp-echo), was measured according to current recommendations (Figure 1). VE-MRI was performed on a 1.5 or 3.0 Tesla MRI system during free breathing, with single-direction in-plane velocity-encoding in phase-encoding direction, angulated parallel to the LV inflow direction. Velocity sensitivity Venc was set to 30cm/s and scan parameters were: spatial resolution 2.5×2.5×8.0 mm^3^, flip angle 10°, echo-time 3.4-3.8ms and repetition-time 5.4-5.9ms. Local LV inflow velocity was sampled along a 4cm scan line starting from the tip of the mitral valve and directed into the LV, similarly to the Color M-mode echocardiography; for 11 consecutive sample points equidistantly positioned along the scan line, the point-in-time was assessed when local velocity exceeded 30cm/s (Figure 1). From the position-time relation, Vp was calculated by 2 methods: 1) from the difference quotient between the first and the last sample point (Vp-MRI-DQ) and 2) from linear regression from all sample points (Vp-MRI-LR).

## Results

Good correlation was found between Vp-echo and both Vp-MRI-DQ (r=0.83 p<0.001) and Vp-MRI-LR (r=0.84 p<0.001). For both Vp-MRI-DQ and Vp-MRI-LR, a significant but small underestimation was observed as compared to Vp measured by Color M-mode echocardiography (Vp-MRI-DQ: -7.3±19.7 cm/s, p=0.004; Vp-MRI-LR: -9.9±15.2cm/s, p<0.001), with increasing bias for higher Vp values. Applying age-related cut-off values for Vp to identify elevated LV filling pressures (Table 1), we observed a Kappa-agreement with echocardiography of 0.72, (p<0.001) for Vp-MRI-DQ and 0.69 (p<0.001) for Vp-MRI-LR.

**Table 1:** Weighted-kappa agreement between Vp-echo and MRI Vp-MRI-DQ and Vp-MRI-LR.

		Vp-MRI-DQ		Vp-MRI-LR	
	
		< 45/55 cm/s	≥ 45/55 cm/s	< 45/55 cm/s	≥ 45/55 cm/s
Vp-echo	< 45/55 cm/s	23	6	23	7
	
	≥ 45/55 cm/s	3	33	3	32

Age-related cut-off values were used to classify elevated LV filling pressures (Vp<45 cm/s for subjects older than 30 years and Vp<55 cm/s for subjects younger than 30 years old).

## Conclusions

High-temporal VE-MRI represents a novel approach to assess Vp showing good correlation with Color M-mode echocardiography. In healthy subjects and patients with heart failure, this new method demonstrated good agreement with echocardiography to identify elevated LV filling pressures.

## Funding

E.E. Calkoen is financially supported by a grant from the Willem-Alexander Kinder- en Jeugdfonds, J.J.M. Westenberg is financially supported by a grant from the Dutch Technology Foundation (STW), project number 11626

